# Interpreting [^18^F]FDG-avid fractures after induction therapy in multiple myeloma: insights from the PET/CT sub-study of the Phase 3 GMMG-HD7 trial

**DOI:** 10.1007/s00259-026-07962-8

**Published:** 2026-06-03

**Authors:** Christos Sachpekidis, Marina Hajiyianni, Markus Wennmann, Annette Kopp-Schneider, Lukas John, Sandra Sauer, Ulrike Dapunt, Niels Weinhold, Ekaterina Menis, Anna Jauch, Uta Bertsch, Michael Hundemer, Alexander Brobeil, Jens Hillengass, Marc S. Raab, Elias K. Mai, Antonia Dimitrakopoulou-Strauss, Hartmut Goldschmidt

**Affiliations:** 1 Clinical Cooperation Unit Nuclear Medicine, German Cancer Research Center (DKFZ), Im Neuenheimer Feld 280, 69210 Heidelberg, Germany; 2 Heidelberg Myeloma Center, Internal Medicine V, Hematology, Oncology and Rheumatology, Heidelberg University Hospital, Heidelberg, Germany; 3 Division of Radiology, German Cancer Research Center (DKFZ), Heidelberg, Germany; 4 Division of Biostatistics, German Cancer Research Center (DKFZ), Heidelberg, Germany; 5 Institute of Human Genetics, University of Heidelberg, Heidelberg, Germany; 6 Institute of Pathology, Heidelberg University Hospital, Heidelberg, Germany; 7 Department of Medicine-Myeloma, Roswell Park Comprehensive Cancer Center, Buffalo, NY USA; 8 National Center of Tumor Diseases Heidelberg, Heidelberg, Germany

**Keywords:** Multiple myeloma, [^18^F]FDG PET/CT, Induction therapy, Treatment response evaluation, Fractures

To the Editor:

[^18^F]FDG PET/CT is increasingly used to assess treatment response in multiple myeloma (MM), and metabolic complete response after first-line therapy is associated with favorable outcome. Interpretation of post-induction scans may, however, be complicated by newly detected [^18^F]FDG-avid fractures, a finding for which specific guidance remains limited. We therefore examined this imaging pattern in the PET/CT sub-study of the phase 3 GMMG-HD7 trial. In a small, highly selected cohort of patients who achieved complete metabolic remission of all baseline myeloma lesions after induction therapy, newly detected [^18^F]FDG-avid fractures were not associated with disease progression during follow-up, supporting the interpretation that these lesions most likely reflect fracture healing rather than residual active myeloma.

[^18^F]FDG PET/CT plays a key role in staging, prognostic stratification, and response assessment in MM. PET negativity during and after first line therapy is associated with improved progression-free survival as well as overall survival and is therefore considered a clinically meaningful milestone in treatment evaluation [1–3]. However, interpretation can be challenging in the post-therapy setting, due to recognized sources of false-positive uptake.

Pathological fractures are common in MM, are associated with advanced skeletal involvement and contribute substantially to morbidity, impaired quality of life and adverse prognosis [4–6]. Fractures exhibit increased [^18^F]FDG uptake during both the acute and reparative phases, which persist for several weeks and complicate treatment response interpretation [7]. While contemporary PET-based response frameworks, including the Italian Myeloma criteria for PET Use (IMPeTUs), acknowledge fractures as skeletal findings -primarily on the CT component- specific guidance regarding associated [^18^F]FDG uptake remains limited [8]. Consensus recommendations emphasize that recent fractures show increased [^18^F]FDG uptake and should be considered carefully when attributing residual metabolic activity to active disease [7].

Against this background, the clinical significance of newly detected [^18^F]FDG-avid fractures on post-induction PET/CT in MM remains unclear. We therefore report a series of transplant-eligible patients with newly diagnosed MM treated in the prospective randomized phase III GMMG-HD7 trial (NCT03617731) [9, 10] who demonstrated this imaging pattern, and we explore its association with clinical outcome. All patients completed induction therapy followed by high-dose melphalan (HDT) and autologous stem cell transplantation (ASCT) and subsequently received maintenance therapy.

Patients were identified who had undergone [^18^F]FDG PET/CT, according to protocol-defined timepoints, at baseline and after induction therapy with lenalidomide-bortezomib-dexamethasone (RVd) or isatuximab-lenalidomide-bortezomib-dexamethasone (Isa-RVd). Inclusion criteria for the current analysis were complete metabolic remission of all baseline myeloma-related lesions on post-induction PET/CT, according to established PET-based response criteria [2], together with the presence of one or more new [^18^F]FDG-avid skeletal lesions interpreted as fractures based on anatomical localization and CT findings. PET/CT acquisition protocols and response criteria have been reported previously [11]. Given the exploratory nature and small sample size, analyses were descriptive.

Among 75 patients with available baseline and post-induction [^18^F]FDG PET/CT, six fulfilled the inclusion criteria. The median follow-up of the entire cohort from the time of post-induction PET/CT was 45.9 months (95% CI: 41.6–48.6 months), and 39.7 months (95% CI: 37.6 months – not reached) for the six patients included in this analysis. In all six cases, baseline myeloma-related lesions showed complete metabolic remission, and no additional sites suspicious for residual or recurrent disease were identified. Newly detected [^18^F]FDG-avid fractures were partly observed at sites of previous osteolytic lesions. None of the six patients experienced disease progression during follow-up (20 progression events occurred in the overall study population at the time of analysis). These observations suggest that fracture-related [^18^F]FDG uptake detected at post-induction PET/CT was not associated with relapse in this selected cohort. The main demographic and clinical characteristics of patients with newly identified [¹⁸F]FDG-avid fractures on PET/CT after induction therapy, including post-induction IMWG response and bone marrow MRD status assessed by next-generation flow cytometry (sensitivity 10^− 5^), are provided in Table 1. Corresponding imaging characteristics, assessed according to the Italian Myeloma Criteria for PET Use (IMPeTUs) [8], including features of post-induction fractures, are presented in Table 2, together with a representative imaging example in Fig. 1.

**Table 1:** Main demographic and clinical characteristics of patients with newly identified [¹⁸F]FDG-avid fractures on PET/CT after induction therapy, without evidence of residual metabolically active disease elsewhere. Progression-free survival (PFS) was calculated from the time of post-induction PET/CT

	Age	Sex	ISS	High-risk cytogenetics	Elevated LDH	*R*-ISS	Induction treatment	IMWG response after induction	MRD status after induction (10^− 5^)	PFS (months)
Patient 1	57	M	I	no	yes	II	Isa-RVd	MR	negative	38.0
Patient 2	50	M	I	no	no	I	RVd	nCR	negative	42.1
Patient 3	46	F	I	no	no	I	Isa-RVd	PR	positive	41.4
Patient 4	57	M	I	no	no	I	RVd	PR	positive	37.6
Patient 5	66	M	II	no	no	II	RVd	PR	negative	36.0
Patient 6	70	F	III	no	no	II	RVd	CR	negative	46.3

*M*, male; *F*, female; *ISS*, international staging system; *LDH*, lactate dehydrogenase; *R-ISS*, revised international staging system; *IMWG*, international myeloma working group; *MRD*, minimal residual disease; *PFS*, progression-free survival; *Isa-RVd*, isatuximab-lenalidomide-bortezomib-dexamethasone; *RVd*, lenalidomide-bortezomib-dexamethasone; *MR*, minimal response; *nCR*, near complete response; *PR*, partial response; *CR*, complete response

**Table 2:** Imaging characteristics of patients with newly identified [¹⁸F]FDG-avid fractures on PET/CT after induction therapy, without evidence of residual metabolically active disease elsewhere. Imaging findings were assessed according to the Italian Myeloma Criteria for PET Use (IMPeTUs) [8]. The localization and highest SUVmax of post-induction fractures are also reported

	Baseline PET/CT						Post-induction PET/CT							
	DS BM	DS FL	Fx	Lx	PMD	EMD	DS BM	DS FL	Fx	Lx	PMD	EMD	Localization new fractures	Highest SUVmax fractures
Patient 1	4	1	1	4	neg	pos	3	1	1	4	neg	neg	Clavicle	6.6
Patient 2	4	5	4	4	pos	neg	3	3	1	4	neg	neg	Clavicle, sternum	5.0
Patient 3	4	4	2	4	neg	neg	3	3	1	4	neg	neg	Ribs, sternum	4.8
Patient 4	3	4	4	4	pos	neg	3	3	1	4	neg	neg	Rib	3.0
Patient 5	3	5	4	4	pos	neg	3	3	1	4	neg	neg	Iliac bone	4.0
Patient 6	4	4	2	2	neg	neg	3	3	1	3	neg	neg	Ribs	8.1

*DS BM*, Deauville score in the bone marrow (score, 1–5); *DS FL*, Deauville score in the hottest focal, [^18^F]FDG-avid, medullary lesion (score, 1–5); *Fx*, number of focal, [^18^F]FDG-avid medullary lesions (score, 1–4); *Lx*, number of lytic lesions (score, 1–4); *PMD*, paramedullary disease; *EMD*, extramedullary disease; *SUVmax*, maximum standardized uptake value; *pos*, positive; *neg*, negative

**Fig. 1:**
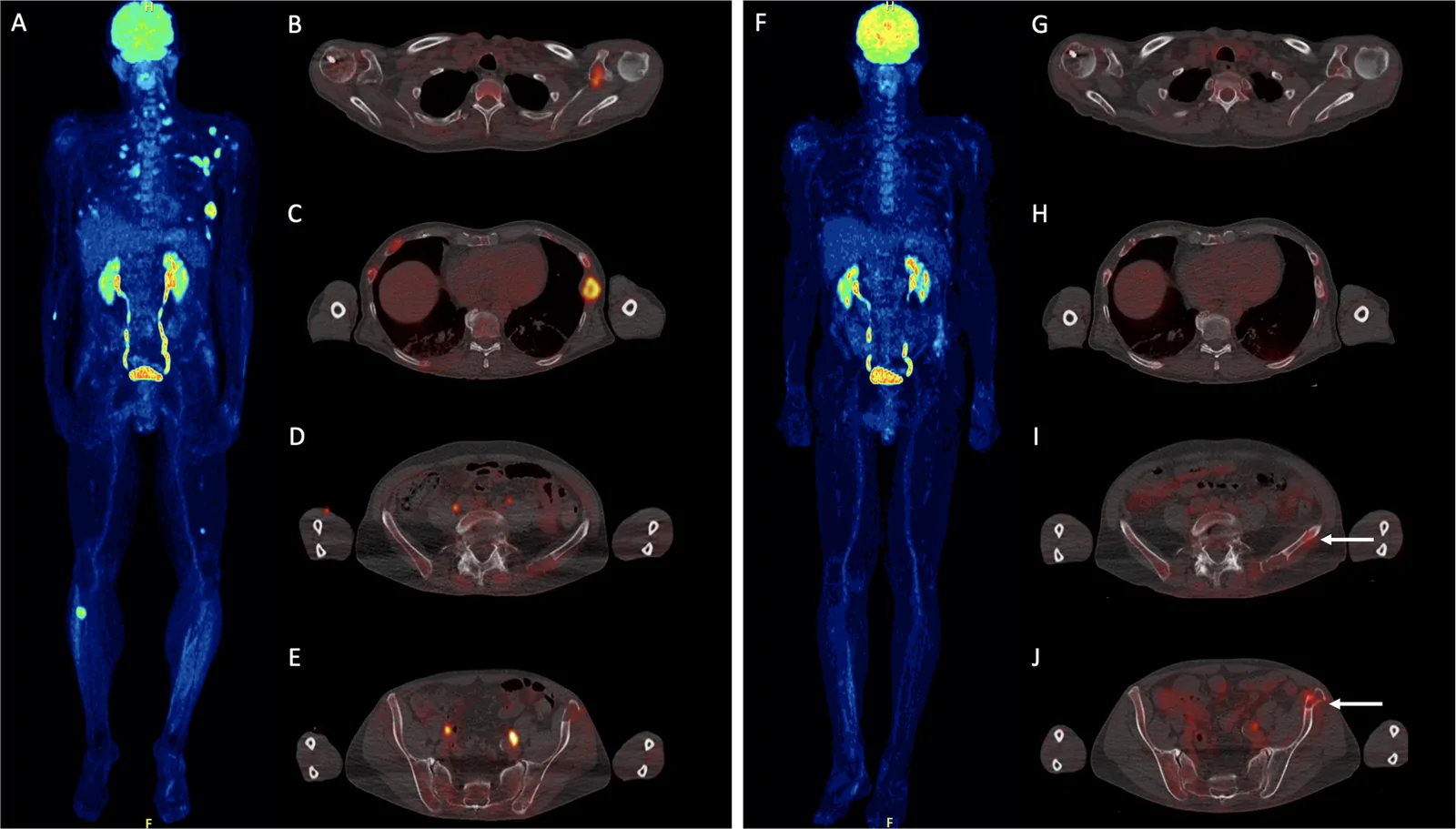
[^18^F]FDG PET/CT of a 66-year old male patient with MM, ISS stage II and R-ISS stage II, before (**A**-**E**) and after (**F**-**J**) induction treatment with lenalidomide, bortezomib, and dexamethasone (RVd). At baseline, PET/CT demonstrated multiple intensely hypermetabolic focal myeloma lesions, including involvement of the left scapula (**B**) and ribs, partly associated with soft tissue extension consistent with paramedullary disease (PMD) (**C**). In addition, lytic lesions with mild [^18^F]FDG uptake were observed, for example in the left iliac bone (**D**, **E**). Following induction therapy, the patient achieved a partial response according to IMWG criteria, and achieved MRD negativity. All focal myeloma manifestations showed complete metabolic remission according to IMPeTUs criteria (**G**, **H**). Concurrently, two new [^18^F]FDG-avid fractures developed in the left iliac bone (**I**), partly at the site of pre-existing osteolysis (**J**) (arrows). At the time of analysis, the patient had not shown disease progression and had achieved a PFS of 36.0 months

This analysis addresses an important interpretative challenge in [^18^F]FDG PET/CT response assessment in MM: the occurrence of new metabolically active fractures in patients otherwise achieving complete metabolic remission after induction therapy. Our observations showed that such findings do not reflect residual or recurrent myeloma.

[^18^F]FDG uptake in fractures is a well-recognized consequence of inflammatory activity and bone repair [7]. In MM, where skeletal fragility and fracture risk are common, this phenomenon is particularly relevant following effective systemic therapy. Accurate interpretation of post-induction PET/CT is critical, as PET response at this time point is highly prognostic and increasingly used to guide treatment decisions in both clinical trials and routine practice [12–15].

Although fractures are acknowledged in the IMPeTUs criteria, they are primarily considered CT-based findings, and no specific recommendations are provided regarding interpretation of associated metabolic activity [8]. Furthermore, to our knowledge, no data are available regarding the frequency of post-therapy fractures in this setting. As a result, [^18^F]FDG-avid fractures may be misclassified as persistent disease, potentially leading to inappropriate response categorization or treatment escalation [7]. Our observations provide preliminary evidence that the development of [^18^F]FDG-avid fractures on post-induction PET/CT, in the context of complete metabolic remission elsewhere, does not appear to be associated with early disease progression.

The main limitation of this analysis is the small number of cases, reflecting the stringent selection of a specific imaging phenotype. In addition, the present report focuses on the post-induction time point; longer-term outcome data across subsequent treatment phases are ongoing. Accordingly, these findings should be analyzed in a larger patient cohort followed for a longer period.

In conclusion, [^18^F]FDG-avid fractures detected on post-induction PET/CT in MM likely do not indicate persistent active myeloma at synchronous remission of other disease manifestations. Instead, the [^18^F]FDG-avidity is rather the result from ongoing fracture healing. Awareness of this interpretative challenge may help avoid misclassification of metabolic response and support more accurate, clinically meaningful use of PET/CT in MM.

## Data Availability

The datasets generated during and/or analysed during the current study are available from the corresponding author on reasonable request.
